# Changes in Sleep Regularity and Perceived Life Stress across the COVID-19 Pandemic: A Longitudinal Analysis of a Predominately Female United States Convenience Sample

**DOI:** 10.3390/clockssleep5010001

**Published:** 2022-12-26

**Authors:** Ryan Bottary, Eric C. Fields, Loren Ugheoke, Dan Denis, Janet M. Mullington, Tony J. Cunningham

**Affiliations:** 1 Institute for Graduate Clinical Psychology, Widener University, Chester, PA 19013, USA; 2 Department of Psychology, Westminster College, New Wilmington, PA 16172, USA; 3 Morehouse School of Medicine, Atlanta, GA 30310, USA; 4 Department of Psychology, University of York, York YO10 5DD, UK; 5 Division of Sleep Medicine, Harvard Medical School, Boston, MA 02115, USA; 6 Department of Neurology, Beth Israel Deaconess Medical Center, Boston, MA 02215, USA; 7 Center for Sleep and Cognition, Beth Israel Deaconess Medical Center, Boston, MA 02215, USA

**Keywords:** age, chronotype, COVID-19, Munich chronotype questionnaire, social jetlag, social sleep restriction

## Abstract

The Coronavirus Disease 2019 (COVID-19) pandemic had a profound impact on sleep and psychological well-being for individuals worldwide. This pre-registered investigation extends our prior study by tracking self-reported social jetlag (SJL), social sleep restriction (SSR), and perceived life stress from May 2020 through October 2021. Using web-based surveys, we collected self-reported sleep information with the Ultrashort Munich Chronotype Questionnaire at three additional timepoints (September 2020, February 2021 and October 2021). Further, we measured perceived life stress with the Perceived Stress Scale at two additional timepoints (February 2021 and October 2021). In a subsample of 181, predominantly female (87%), United States adults aged 19–89 years, we expanded our prior findings by showing that the precipitous drop in SJL during the pandemic first wave (May 2020), compared to pre-pandemic (February, 2020), rapidly rose with loosening social restrictions (September 2020), though never returned to pre-pandemic levels. This effect was greatest in young adults, but not associated with self-reported chronotype. Further, perceived life stress decreased across the pandemic, but was unrelated to SJL or SSR. These findings suggest that sleep schedules were sensitive to pandemic-related changes in social restrictions, especially in younger participants. We posit several possible mechanisms supporting these findings.

## 1. Introduction

Several studies, including participants from across the globe, have demonstrated that the Coronavirus Disease 2019 (COVID-19) pandemic resulted in a shift in psychological well-being [1,2,3] and sleep behavior [4]. Among the many pandemic-related concerns, risk of infection, associated serious medical outcomes, and imposed social restrictions were linked to increased stress and poorer psychological well-being [5,6]. At the same time, sleep patterns changed in response to early pandemic-related social restrictions and included, compared to pre-pandemic, greater sleep timing regularity (i.e., reduced social jetlag or “SJL”) and/or sleep duration regularity (i.e., reduced social sleep restriction or “SSR”) [7,8,9,10,11,12,13,14]. In particular, changes in SJL (i.e., the difference in sleep midpoint on workdays and free days; [15]) and SSR (i.e., the difference in sleep duration on workdays and free days; [8]) were hypothesized to have been impacted as the result of increased occupational, educational and social schedule flexibility during the pandemic, potentially affording individuals the opportunity to sleep in greater attunement with their intrinsic circadian rhythms. For example, several reports have provided evidence that younger individuals, as well as those with an evening chronotype—two groups susceptible to variable regularity in sleep timing and duration due to social-biological sleep desynchrony [15,16]—demonstrated the greatest reductions in SJL and SSR early in the pandemic compared to pre-pandemic [7,8,10].

Sleep quality and psychological well-being are highly interrelated [17,18]. Thus, it is possible that observed improvements in sleep timing regularity may have helped buffer the impact of pandemic-related worries on psychological distress. In fact, self-reported evening chronotype [19,20] and increased SJL [21,22,23], have been linked to poorer mental health and reduced emotional regulation. Our prior report [7] showed that those exhibiting the greatest reductions in SJL and SSR from pre-pandemic to the first wave of the pandemic also reported the lowest perceived stress during the first wave of the pandemic. Interestingly, perceived life stress was highest in younger participants and those with an evening chronotype. However, we did not have pre-pandemic ratings of perceived life stress, thus were limited in making claims about potential associations between changes in sleep regularity and changes in perceived life stress.

To date, there remains a lack of longitudinal studies measuring regularity of sleep timing and duration (i.e., SJL and SSR, respectively) and psychological distress during the full course of the COVID-19 pandemic, including pivotal milestones such as vaccine roll-outs and re-openings of occupational, educational, and social settings. The present investigation follows-up on our previous study [7] by aiming to determine whether pandemic-associated reductions in SJL and SSR in United States (US) participants have since returned to baseline levels with loosening of pandemic-imposed social restrictions. Further, we investigated whether such effects varied with respect to age and self-reported chronotype. Finally, as sleep regularity may be key for optimal mental health (which may be adversely impacted by life stress; [24]), we assessed whether changes in sleep regularity across the additional time-points tracked with changes in perceived life stress.

We hypothesized that the initial reductions in SJL and SSR observed during the first-wave of the COVID-19 pandemic, compared to pre-pandemic, would not be sustained across additional time points, when social restrictions were lifted. We further hypothesized that this reversal would be most pronounced in younger adults and those with an evening chronotype. Finally, we predicted that increases in SJL and SSR from the pandemic first-wave to additional time points would be associated with greater increases in perceived stress.

## 2. Results

Descriptive statistics for all measures are listed in Table S2.

### 2.1. Social Jetlag

**Figure 1:**
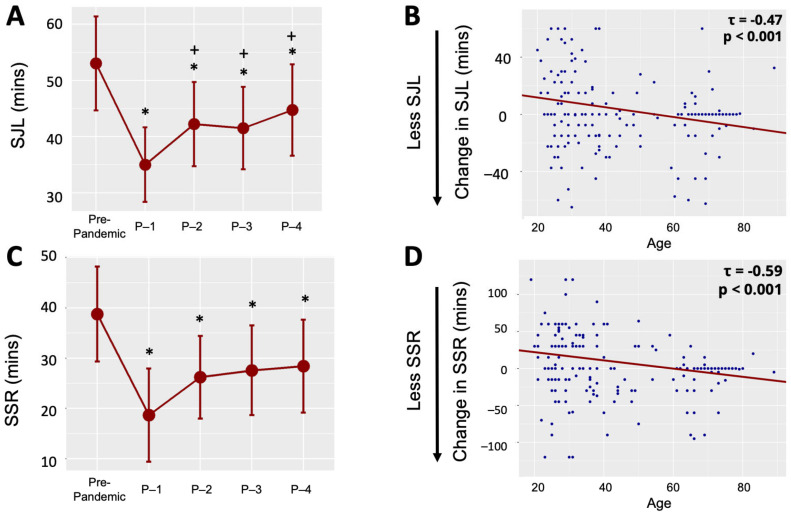
Changes in (**A**) social jetlag (SJL; free day—workday sleep midpoint) and (**B**) social sleep restriction (SSR; free day—workday sleep duration) across the COVID-19 pandemic. Dots represent trimmed means. Error bars show the confidence interval of the difference. Timepoints on x-axis reflect µMCTQ survey release dates: *Pre-Pandemic*, 1 February 2020; *Pandemic-1 (P-1)*, 19 May 2020; *Pandemic-2 (P-2)*, 28 September 2020; *Pandemic-3 (P-3)*, 27 February 2021; *Pandemic-4 (P-4)*, 28 October 2021. Younger age is associated with greater increases in (**C**) SJL and (**D**) SSR following the pandemic first wave. The y-axis represents the change in SJL or SSR, respectively, between Pandemic-1 (i.e., averaged across the six weeks prior to 19 May 2020) and Pandemic-2 (i.e., averaged across the six weeks prior to 28 September 2020). For visualization purposes, outliers, defined as values more than three median absolute deviations (scaled to estimate the standard deviation) from the median, were excluded (but were not excluded in analyses, which instead used statistical approaches that are robust to outliers). The line of best fit was estimated via Theil-Sen regression. *, statistically significant reduction from Pre-Pandemic; +, statistically significant increase from P-1.

For sleep midpoint, the Time Point × Day ANOVA revealed main effects of Time Point, Q = 9.01, *p* < 0.001, and Day, Q = 189.6, *p* < 0.001, as well as a Time Point × Day interaction, Q = 6.67, *p* < 0.001. Follow-up tests revealed that free-day sleep midpoint was later than workday sleep midpoint across all five µMCTQ time points (all ps < 0.001, see Table S3). SJL was reduced at all pandemic time points compared to pre-pandemic (all ps < 0.02, see Figure 1A and Table S3). Conversely, SJL was significantly greater at Pandemic 2, 3 and 4 compared to Pandemic 1 (all ps < 0.05, see Figure 1A and Table S3). This suggests that SJL (1) sharply decreased from Pre-Pandemic to Pandemic-1, (2) sharply increased from Pandemic-1 to Pandemic-2, (3) but never fully returned to Pre-Pandemic levels. We previously reported the relationships between age, self-reported chronotype, and the change in SJL from Pre-Pandemic to Pandemic-1 [7]. Therefore, our regression model for age and chronotype included change in SJL from Pandemic-1 to Pandemic-2, the timepoints in which the reversal in SJL first became apparent and after which it appeared to plateau (see Figure 1A). The regression model revealed that change in SJL from Pandemic-1 to Pandemic-2 was associated with age when controlling for rMEQ score, b = −0.47, 95% CI [−0.92, −0.16], *p* < 0.001 (see Figure 1B), suggesting that the youngest participants demonstrated the greatest increases in SJL. However, SJL was not associated with rMEQ when controlling for age, b = −0.09, 95% CI [−1.40, 1.04], *p* = 0.851.

### 2.2. Social Sleep Restriction

For sleep duration, a Time Point × Day ANOVA revealed no main effect of Time Point, Q = 1.69, *p* = 0.15, but a main effect of Day, Q = 70.93, *p* < 0.001, and a Time Point × Day interaction, Q = 4.54, *p* = 0.001. Follow-up tests revealed that free-day sleep duration was longer than workday sleep duration across all five timepoints (all ps < 0.001, see Table S3). Further, SSR was reduced at all timepoints compared to pre-pandemic (all ps < 0.02, see Figure 1C and Table S3). SSR did not significantly increase from Pandemic-1 to any of the subsequent Pandemic time points (i.e., 2–4, ps > 0.08). This suggests that SSR (1) sharply decreased from Pre-Pandemic to Pandemic-1, (2) increased numerically, but not significantly from Pandemic-1 to Pandemic-2, and (3) never fully returned to Pre-Pandemic levels. Although SSR did not significantly increase from Pandemic-1 to Pandemic-2, regression analyses with age and self-reported chronotype parallel those of SJL (see Figure 1D and Table S4).

### 2.3. Perceived Life Stress Changes and Associations with Age, Self-Reported Chronotype, and Sleep Regularity Measures

Trimmed means t-tests revealed that PSS scores decreased from Pandemic-1 (Mt (Mt, 20% trimmed mean) = 18.7, sw* (sw*, 20% winsorized standard deviation (scaled to estimate the standard deviation under normality) = 7.72) to Pandemic-3 (Mt = 14.89, sw* = 8.48), t(99.0) = −7.12, *p* < 0.001, and Pandemic-4 (Mt = 14.99, sw* = 8.86), t(99.0) = −6.55, *p* < 0.001. However, PSS scores did not significantly differ from Pandemic-3 to Pandemic-4, t(99.0) = 0.18, *p* = 0.86.

As these tests revealed that PSS scores changed significantly from Pandemic-1 to Pandemic-3, but not Pandemic-3 to pandemic-4, we calculated a PSS change score using the former two time points (i.e., Pandemic-3*—*Pandemic-1). We then correlated this PSS change score with SJL and SSR change scores calculated using the Pandemic-1 and Pandemic-3 µMCTQ time points (i.e., Pandemic-3*—*Pandemic-1), which best aligned with the PSS time points. Change in PSS from Pandemic-1 to Pandemic-3 was not associated with age (τ = 0.00, 95% CI [−0.10, 0.11], *p* = 0.92), self-reported chronotype (τ = 0.08, 95% CI [−0.02, 0.17], *p* = 0.16), or change in SJL (τ = −0.00, 95% CI [−0.11, 0.10], *p* = 0.94) or SSR (τ = −0.01, 95% CI [−0.11, 0.10], *p* = 0.88) from Pandemic-1 to Pandemic-3. These findings suggest that while perceived life stress decreased from Pandemic-1 to Pandemic-3, this decrease was not associated with the co-occurring changes in sleep regularity.

## 3. Discussion

The present study is, to our knowledge, the first to investigate changes in sleep regularity and perceived life stress across a nearly two-year period during the COVID-19 pandemic. Consistent with our hypotheses, initial decreases in SJL and SSR during the first wave of the pandemic (i.e., during the 6 weeks prior to 19 May 2020), compared to pre-pandemic (i.e., during the 6 weeks prior to 1 February 2020), were partially reversed later the same year (i.e., during the 6 weeks prior to 28 September 2020), when social restrictions were mostly lifted. This reversal was most pronounced in the youngest participants, though was not associated with self-reported chronotype, and increased in magnitude through the latest collected time point (i.e., during the 6 weeks prior to 28 October 2021], but never returned to pre-pandemic levels. Interestingly, perceived life stress decreased across the pandemic, though this change was not associated with changes in regularity of sleep timing (i.e., SJL) or duration (i.e., SSR).

With respect to SJL and SSR, we interpret the current findings to suggest that changes in sleep regularity resulted from imposition, and subsequent lifting, of social restrictions implemented in response to the COVID-19 pandemic. Specifically, we posit that initial reductions in SJL and SSR during the first wave of the pandemic, compared to pre-pandemic, were the result of social restrictions including stay-at-home orders and temporary closure of occupational, educational and social settings. These restrictions and closures, in turn, may have allowed for increased flexibility of sleep scheduling and perhaps maintaining sleep timing more attuned with intrinsic circadian rhythms. Conversely, our data suggest that SJL and SSR sharply increased in parallel with the lifting of social restrictions. Further, both SJL and SSR continued to marginally increase with the progressive relaxation of social restrictions and a return to occupational, educational, and social commitments. However, SJL and SSR did not return to pre-pandemic levels at our latest studied time point (i.e., during the 6 weeks prior to 28 October 2021). While we can only speculate on the cause of this, the normalization of remote and hybrid work models during the pandemic may have been one factor contributing to the attenuation of SJL and SSR compared to pre-pandemic, when such practices were rare [25,26].

Our findings suggest that perceived life stress decreased across the pandemic. Habituation to the ongoing threat of infection, vaccine availability, and more flexible work scheduling are all potential contributors, though we can only speculate on this as our current dataset did not explicitly measure these variables. Contrary to our expectations, changes in SJL and SSR were not related to changes in perceived life stress. However, sleep regularity is only one marker of sleep quality. In fact, meta-analytic evidence suggests that self-reported poor sleep, quantified using measures such as the Pittsburgh Sleep Quality Index and Insomnia Severity Index, decreased from the early pandemic, when social restrictions were instituted, to later in the pandemic, when such restrictions were lifted [27]. Future work, drawing from other prospective datasets, might help to clarify if other measures of sleep quality might hold greater predictive value for determining changes in psychological well-being across the pandemic.

### Limitations

It is important to note the limitations of the current report. By including only participants that completed all follow-up assessments, our sample size was reduced significantly compared to our previous report [7]. However, we replicated, in this smaller sample, the original findings demonstrating a decrease in SJL and SSR from pre-pandemic to the pandemic first-wave. Further, the sample in the current study consisted predominantly of well-educated, white, non-Hispanic, cis-gender women. Thus, these findings may not necessarily reflect the US population at large, and the surprisingly heavy skew of the sample toward women (87%) in particular prevented meaningful follow-up analyses on the effects of sex on these relationships. Lastly, many important socio-political events occurred in parallel with the COVID-19 pandemic [28], making isolation of specific factors contributing to sleep and psychological well-being difficult.

## 4. Materials and Methods

### 4.1. Data Collection

This investigation was pre-registered on Open Science Framework (OSF; https://osf.io/ud6ch/). Data were collected between 20 March 2020 and 7 December 2021. Analyses were conducted on a subset of sleep and psychological measures from a web-based, longitudinal, observational study investigating changes in sleep and emotional wellbeing during the COVID-19 pandemic [2,29,30]. The larger study was open globally to English-speaking individuals ≥18 years of age with access to technology. Participants were recruited through word of mouth, social media posts, and snowballing methods. All survey data were collected online using the Research Electronic Data Capture (REDCap™) system [31,32] through Boston College. For a full description of the larger study, see references [2,30] or protocols posted to our main study OSF page (https://osf.io/gpxwa/).

### 4.2. Participants

Analyses for the present investigation were conducted on a subset of 181 participants, aged 19–89 years, who met the following inclusion criteria: (1) resided in the US during the data collection period, (2) were not engaged in shift work at any of the data collection timepoints, and (3) completed all sleep and psychological questionnaires of interest to the present investigation at all of the designated timepoints.

Participant demographics are listed in Table S1. This investigation follows-up on a previously published report [7]. Consistent with this original report, we restricted our analyses to US residents due to differing timelines of lockdowns, social restrictions, and pandemic severity across the globe. Ethical approval for the larger study was obtained from the Boston College Institutional Review Board and all participants completed informed consent prior to study participation. As compensation, participants received entries into raffles to receive gift cards.

### 4.3. Assessments

#### 4.3.1. Demographics Survey

Upon enrollment in the larger study [30] (see https://osf.io/gpxwa/), participants completed a demographics survey that queried several participant characteristics including age, biological sex, gender identity, race, ethnicity, education level, and socioeconomic status (see Table S1).

#### 4.3.2. Ultrashort Munich Chronotype Questionnaire (µMCTQ)

The µMTCQ [33] queried participants’ work and sleep behaviors in the 6 weeks prior to its administration at each time point. Participants were asked if they had engaged in shift-work, the average number of days per week they worked, and their average sleep start and sleep end times on work days and free days. Based on µMCTQ responses, we determined sleep duration (i.e., total amount of time between sleep-onset time and sleep-end time) and sleep midpoint (circular mean of sleep-onset and sleep-end times expressed as minutes past midnight) on workdays and free days. We further calculated social jetlag (i.e., the difference between workday and free-day sleep midpoint [15] and social sleep restriction (i.e., the difference between workday and free-day sleep duration [8].

#### 4.3.3. Reduced Morningness-Eveningness Questionnaire (rMEQ)

The rMEQ [34] is a 5-item version of the Morningness-Eveningness Questionnaire [35]. The rMEQ asks for respondents to report perceived optimal bed- and rise-times, tiredness upon waking from sleep, the time of day when respondents feel their best, and whether respondents consider themselves to be a “morning” or “evening” type. Scores range from 4–26, with a score of <12 indicating an evening type, 12–17 a neither type and >17 a morning type.

#### 4.3.4. Perceived Stress Scale (PSS)

The PSS [36] assesses respondent’s feelings and perceived stress over the previous month. Scores range from 0–40, with scores ≤13 indicating low perceived stress, 14–26 indicating moderate perceived stress, and 27–40 indicating high perceived stress.

### 4.4. Procedures

During study enrollment, which began on 20 March 2020, participants completed the demographics survey. Participants later completed the µMCTQ on four separate occasions. For the first administration, participants completed the µMCTQ twice at one time, querying sleep patterns in the 6-weeks prior to 1 February 2020 (Pre-Pandemic) and in the 6-weeks prior to 19 May 2020 (Pandemic-1). The µMCTQ was then used to query sleep patterns in the 6-weeks prior to three additional time points: (1) 28 September 2020 (Pandemic-2), (2) 27 February 2021 (Pandemic-3), and (3) 28 October 2021 (Pandemic-4). This resulted in a total of 5 µMCTQ time points (i.e., Pre-Pandemic and Pandemic 1–4). The rMEQ was administered separately to participants on 16 June 2020. Finally, we collected the PSS at three timepoints, 16 June 2020 (Pandemic-1), 27 February 2021 (Pandemic-3), and 28 October 2021 (Pandemic-4).

### 4.5. Statistical Analyses

Due to skew and outliers in the sleep data, which limit the validity of statistical models that assume normality, we compared sleep midpoint and sleep duration across study time points using a robust analysis of variance (ANOVA) based on trimmed means [37]. This test is robust to outliers and to a broad range of distributions both in terms of Type I error rate and power. Analysis of sleep midpoint and sleep duration was conducted using a 5 Time Point (Pre-Pandemic, Pandemic-1, Pandemic-2, Pandemic-3, Pandemic-4) × 2 Day (Workday, Free-day) trimmed means ANOVA testing the main effect of the pandemic phase, the main effect of Workday vs. Free-Day sleep, and their interaction. Follow-up trimmed t-tests with confidence intervals for the difference in trimmed means were conducted comparing Pre-Pandemic and Pandemic-1 SJL and SSR to SJL and SSR at each subsequent pandemic time point (i.e., Pandemic 2–4). PSS scores at each collected time point (i.e., Pandemic-1, Pandemic-3, Pandemic-4) were compared using trimmed means t-tests.

We correlated age, self-reported chronotype, SJL, SSR and PSS using Kendall’s Tau correlations. Consistent with our previous report [7], we observed a significant correlation between age and rMEQ scores, τ = 0.20, 95% CI [0.09, 0.30], *p* < 0.001. Because of this, we performed robust regression analyses. To predict changes in SJL and SSR based on both age and rMEQ scores, controlling for the other, we used the Theil-Sen estimator with (1) the Harrell-Davis estimate of the population median in place of the sample median in the Theil-Sen algorithm, (2) the Gauss-Seidel method for extending Theil-Sen to multiple predictors, and (3) a percentile bootstrap calculation of *p*-values and confidence intervals.

## 5. Conclusions

Despite these limitations, we believe the current findings provide a valuable exploration of how sleep schedules change in the presence and absence of socially imposed schedules. One potential implication of these findings is that shifts to remote and hybrid models of work, school, and perhaps even social activities may permit sleep-wake behavior to extend and regularize. This may be especially true for younger individuals for whom sleep timing irregularity, and social jetlag specifically, has been linked to poor physical [38] and mental health outcomes [23]. Whether post-pandemic lifestyle changes will have a positive long-term impact on sleep and mental health remains an open question.

## Data Availability

Data from the reported analyses is available on Open Science Framework: https://osf.io/gpxwa/.

## Supplementary Materials

The following supporting information can be downloaded at: https://www.mdpi.com/article/10.3390/clockssleep5010001/s1; Table S1: Participant Demographics; Table S2: Descriptive statistics; Table S3: Analysis of sleep midpoint and sleep duration; Table S4: Age and chronotype regression models.
