# Supplementary material for: Changes in Sleep Regularity and Perceived Life Stress across the COVID-19 Pandemic: A Longitudinal Analysis of a Predominately Female United States Convenience Sample
Source: Clocks Sleep. 2022 Dec 26;5(1):1–9. doi: 10.3390/clockssleep5010001 (PMC9844449; doi:10.3390/clockssleep5010001)
Supplement: Supplementary file 1 [file clockssleep-05-00001-s001.zip › clockssleep-2065455-supplementary.pdf]

**Supplementary Materials**

**Changes in Sleep Regularity and Perceived Life Stress Across the COVID-19 Pandemic: A Longitudinal Analysis of a Predominately Female United States Convenience Sample**

Ryan Bottary, Eric C. Fields, Loren Ugheoke, Dan Denis, Janet M. Mullington, Tony J. Cunningham

**Table S1**. Participant Demographics.

| | |
|---|---|
| **N** | 181 |
| **Age** | |
| Mean | 42.29 |
| Standard Deviation | 18.51 |
| Minimum | 19 |
| 25% | 27 |
| 50% | 34 |
| 75% | 61 |
| Maximum | 89 |
| **Ethnicity** | |
| Hispanic | 1.7% |
| Not Hispanic | 97.2% |
| Prefer not to say | 1.1% |
| **Race** | |
| African American | 1.7% |
| Asian | 6.6% |
| White | 85.6% |
| Hispanic/Latinx | 0.6% |
| Native Hawaiian or other Pacific Islander | 0.0% |
| American Indian/Alaska Native | 0.0% |
| More than one race/prefer to self-describe | 5.0% |
| Unknown | 0.0% |
| Prefer not to say | 0.6% |
| **Gender** | |
| Female | 84.0% |
| Male | 12.7% |
| Non-binary/third gender | 1.7% |
| Prefer to self-describe | 0.0% |
| Prefer not to say | 0.6% |
| Not reported | 1.1% |
| **Biological Sex** | |
| Female | 87.3% |
| Male | 12.7% |
| **Gender Identity** | |
| Transgender | 1.1% |

| | |
|---|---|
| Cisgender | 97.8% |
| Prefer not to say | 1.1% |
| Not reported | 0.0% |
| **Sexual Orientation** | |
| Gay/lesbian | 4.4% |
| Bisexual | 11.0% |
| Straight/heterosexual | 82.3% |
| Prefer to self-describe | 1.7% |
| Prefer not to say | 0.6% |
| Not reported | 0.0% |
| **Education** | |
| Some high school | 0.0% |
| High school diploma or GED | 2.2% |
| Some college | 7.7% |
| Bachelor's degree | 22.7% |
| Some post-bachelor | 11.0% |
| Graduate, medical, or professional degree | 56.4% |
| **Relationship Status** | |
| Single | 21.5% |
| In a relationship | 24.3% |
| Married | 45.3% |
| Separated/divorced | 6.1% |
| Widowed | 2.8% |
| **Serious medical problems?** | |
| No | 90.1% |
| Yes | 9.9% |
| **Income** | |
| $0 - 25,000 | 6.1% |
| $25,001 - 50,000 | 14.9% |
| $50,001 - 75,000 | 18.8% |
| $75,001 - 100,000 | 16.0% |
| $100,001 - 150,000 | 19.3% |
| $150,001 - 250,000 | 12.7% |
| $250,000+ | 12.2% |
| not reported | 0.0% |
| **Are you a full time student?** | |
| No | 79.6% |
| Yes | 20.4% |
| **Are you currently employed?** | |
| No | 23.2% |
| Yes | 56.4% |

| | |
|---|---|
| Not reported | 20.4% |
| **Political Ideology** | |
| Very liberal | 35.9% |
| Liberal | 35.4% |
| Slightly liberal | 11.6% |
| Moderate | 11.0% |
| Slightly conservative | 2.2% |
| Conservative | 1.1% |
| Not reported | 2.8% |
| Very conservative | 0.0% |
| **How many dependent children live with you?** | |
| 0 | 83.4% |
| 1 | 8.8% |
| 2 | 6.6% |
| 3 | 1.1% |
| 4 | 0.0% |
| 5+ | 0.0% |
| **How many people do you live with?** | |
| 0 | 16.0% |
| 1 | 43.6% |
| 2 | 17.7% |
| 3 | 12.2% |
| 4 | 6.1% |
| 5+ | 4.4% |
| **U.S. Region** | |
| Northeast | 48.1% |
| South | 11.0% |
| West | 15.5% |
| Midwest | 17.7% |
| Not reported | 7.7% |

**Table S2.** Descriptive statistics.

| | N | M (s) | Mt (sw*) | Md (MADN) | Min | 25th percentile | 75th percentile | Max | skew | kurtosis |
|---|---|---|---|---|---|---|---|---|---|---|
| **Age** | 181 | 42.29 (18.51) | 38.59 (24.12) | 34.00 (13.34) | 19.00 | 27.00 | 61.00 | 89.00 | 0.72 | -0.94 |
| **rMEQ** | 166 | 15.09 (4.54) | 15.13 (4.26) | 15.00 (4.45) | 4.00 | 12.00 | 18.00 | 25.00 | -0.07 | -0.42 |
| **Sleep Midpoint (Hours Past Midnight)** | | | | | | | | | | |
| Pre-Pandemic Workday | 176 | 2.96 (1.18) | 2.85 (.96) | 2.75 (.93) | -0.50 | 2.25 | 3.50 | 8.00 | 0.74 | 2.16 |
| Pre-Pandemic Free-day | 176 | 3.86 (1.41) | 3.73 (1.33) | 3.75 (1.24) | -0.50 | 2.88 | 4.50 | 8.00 | 0.31 | 0.68 |
| Pandemic-1 Workday | 176 | 3.44 (1.53) | 3.30 (1.36) | 3.25 (1.33) | -0.50 | 2.38 | 4.25 | 8.63 | 0.66 | 1.13 |
| Pandemic-1 Free-day | 176 | 4.05 (1.60) | 3.89 (1.48) | 3.75 (1.48) | -0.50 | 3.00 | 5.00 | 8.75 | 0.46 | 0.50 |
| Pandemic-2 Workday | 176 | 3.09 (1.24) | 3.01 (1.07) | 3.00 (1.11) | -0.50 | 2.38 | 3.75 | 8.01 | 0.71 | 1.82 |
| Pandemic-2 Free-day | 176 | 3.78 (1.43) | 3.71 (1.27) | 3.75 (1.27) | -0.50 | 2.86 | 4.50 | 8.00 | 0.33 | 0.53 |
| Pandemic-3 Workday | 176 | 3.06 (1.31) | 2.98 (.98) | 3.00 (.99) | -1.00 | 2.33 | 3.63 | 7.50 | 0.41 | 0.89 |
| Pandemic-3 Free-day | 176 | 3.77 (1.48) | 3.67 (1.21) | 3.62 (1.29) | -1.00 | 2.98 | 4.50 | 7.51 | 0.21 | 0.59 |
| Pandemic-4 Workday | 176 | 3.02 (1.30) | 2.96 (1.06) | 3.00 (1.11) | -1.25 | 2.25 | 3.63 | 8.01 | 0.53 | 1.88 |
| Pandemic-4 Free-day | 176 | 3.80 (1.49) | 3.71 (1.27) | 3.75 (1.30) | -1.25 | 2.75 | 4.50 | 8.00 | 0.15 | 0.61 |
| **Sleep Duration (Hours)** | | | | | | | | | | |
| Pre-Pandemic Workday | 181 | 7.95 (.95) | 7.95 (.91) | 8.00 (.74) | 5.00 | 7.50 | 8.50 | 12.00 | 0.14 | 1.65 |
| Pre-Pandemic Free-day | 181 | 8.55 (1.15) | 8.59 (1.04) | 8.50 (.74) | 4.00 | 8.00 | 9.25 | 12.00 | -0.61 | 2.27 |
| Pandemic-1 Workday | 181 | 8.00 (1.25) | 8.09 (1.10) | 8.00 (1.11) | .00 | 7.50 | 9.00 | 11.75 | -1.52 | 8.57 |
| Pandemic-1 Free-day | 181 | 8.38 (1.37) | 8.40 (1.03) | 8.25 (1.11) | .00 | 7.83 | 9.00 | 15.00 | -0.75 | 9.94 |
| Pandemic-2 Workday | 181 | 7.91 (1.08) | 7.90 (.94) | 8.00 (.99) | 3.00 | 7.25 | 8.50 | 12.50 | -0.06 | 3.41 |
| Pandemic-2 Free-day | 181 | 8.33 (1.17) | 8.34 (0.91) | 8.25 (1.11) | 3.00 | 7.83 | 9.00 | 12.50 | -0.33 | 2.82 |
| Pandemic-3 Workday | 181 | 7.94 (0.98) | 7.97 (1.12) | 8.00 (1.11) | 5.00 | 7.33 | 8.75 | 10.47 | -0.34 | 0.16 |
| Pandemic-3 Free-day | 181 | 8.36 (1.20) | 8.43 (.92) | 8.50 (.74) | .00 | 7.92 | 9.00 | 11.00 | -2.03 | 12.43 |
| Pandemic-4 Workday | 181 | 7.97 (.98) | 7.97 (1.00) | 8.00 (.74) | 5.00 | 7.25 | 8.50 | 11.00 | -0.10 | 0.63 |
| Pandemic-4 Free-day | 181 | 8.47 (1.14) | 8.44 (1.01) | 8.50 (.74) | 5.00 | 8.00 | 9.00 | 12.75 | 0.08 | 1.36 |
| **Perceived Stress Scale** | | | | | | | | | | |
| Pandemic-1 | 166 | 19.04 (7.64) | 18.77 (7.71) | 20.00 (7.41) | 0.00 | 13.00 | 23.75 | 37.00 | 0.10 | -0.29 |
| Pandemic-2 | 180 | 14.91 (7.57) | 14.80 (8.34) | 15.00 (8.90) | 0.00 | 9.00 | 21.00 | 35.00 | 0.16 | -0.61 |
| Pandemic-4 | 180 | 15.31 (7.57) | 15.02 (8.32) | 15.00 (8.90) | 0.00 | 10.00 | 21.00 | 34.00 | 0.23 | -0.58 |

*Note.* rMEQ, Reduced Morningness-Eveningness Questionnaire; M = mean; s = standard deviation; Mt = 20% trimmed mean; sw* = 20% winsorized standard deviation (scaled to estimate the standard deviation under normality); Md = median; MADN = median absolute deviation (scaled to estimate the standard deviation under normality).

**Table S3**. Analysis of sleep midpoint and sleep duration. Bracketed values give 95% confidence intervals. Effect sizes and confidence intervals are in minutes and reflect differences in the trimmed means of the conditions being compared. See text for details of trimmed mean ANOVA and t-test analyses. Significant results bolded for emphasis.

| **Sleep Midpoint** | | | |
|---|---|---|---|
| | *Test Statistic* | *p*-value | Difference (mins) |
| *Trimmed ANOVA* | | | |
| Main effect of Time Point | Q = 9.01 | **<0.001** | |
| Main effect of Day | Q = 189.60 | **<0.001** | |
| Interaction | Q = 6.67 | **<0.001** | |
| *Follow-up trimmed t-tests* | | | |
| Pre-Pandemic Free-day - Workday | t(105.0) = 12.59 | **<0.001** | 53.03 [44.68, 61.38] |
| Pandemic-1 Free-day - Workday | t(105.0) = 10.47 | **<0.001** | 35.03 [28.40, 41.67] |
| Pandemic-2 Free-day - Workday | t(105.0) = 11.19 | **<0.001** | 42.23 [34.75, 49.71] |
| Pandemic-3 Free-day - Workday | t(105.0) = 11.26 | **<0.001** | 41.53 [34.22, 48.84] |
| Pandemic-4 Free-day - Workday | t(105.0) = 10.92 | **<0.001** | 44.75 [36.62, 52.87] |
| *Interaction follow-ups* | | | |
| SJL Pandemic-1 - Pre-Pandemic | Q = 26.82 | **<0.001** | |
| SJL Pandemic-2 - Pre-Pandemic | Q = 9.23 | **0.002** | |
| SJL Pandemic-3 - Pre-Pandemic | Q = 10.18 | **0.001** | |
| SJL Pandemic-4 - Pre-Pandemic | Q = 5.22 | **0.022** | |
| SJL Pandemic-2 - Pandemic-1 | Q = 4.28 | **0.039** | |
| SJL Pandemic-3 - Pandemic-1 | Q = 4.22 | **0.040** | |
| SJL Pandemic-4 - Pandemic-1 | Q = 6.65 | **0.010** | |
| **Sleep Duration** | | | |
| | *Test Statistic* | *p*-value | Difference (mins) |
| *Trimmed ANOVA* | | | |
| Main effect of Time | Q = 1.69 | 0.150 | |
| Main effect of Day | Q = 70.93 | **<.0001** | |
| Interaction | Q = 4.54 | **0.001** | |
| *Follow-up trimmed t-tests* | | | |
| Pre-Pandemic Free-day - Workday | t(108.0) = 8.14 | **<0.001** | 38.77 [29.33, 48.21] |
| Pandemic-1 Free-day - Workday | t(108.0) = 3.99 | **<0.001** | 18.68 [9.40, 27.96] |
| Pandemic-2 Free-day - Workday | t(108.0) = 6.33 | **<0.001** | 26.22 [18.00, 34.44] |
| Pandemic-3 Free-day - Workday | t(108.0) = 6.15 | **<0.001** | 27.60 [18.70, 36.50] |
| Pandemic-4 Free-day - Workday | t(108.0) = 6.10 | **<0.001** | 28.41 [19.18, 37.64] |
| *Interaction follow-ups* | | | |
| SSR Pandemic-1 - Pre-Pandemic | Q = 17.47 | **<0.001** | |
| SSR Pandemic-2 - Pre-Pandemic | Q = 6.72 | **0.010** | |
| SSR Pandemic-3 - Pre-Pandemic | Q = 5.23 | **0.022** | |
| SSR Pandemic-4 - Pre-Pandemic | Q = 4.54 | **0.033** | |
| SSR Pandemic-2 - Pandemic-1 | Q = 2.17 | 0.141 | |
| SSR Pandemic-3 - Pandemic-1 | Q = 2.83 | 0.093 | |
| SSR Pandemic-4 - Pandemic-1 | Q = 3.03 | 0.082 | |

*Note.* SJL, social jetlag; SSR, social sleep restriction.

**Table S4.** Age and chronotype regression models. Results show Theil-Sen regression with the Harrell-Davis estimate of the population median in place of the sample median in the Theil-Sen algorithm and the Gauss-Seidel method for extending Theil-Sen to multiple predictors. Confidence intervals and p-values were calculated via a percentile bootstrap method.

| **Social Jet Lag** (Pandemic-2 - Pandemic-1) | | | | |
|---|---|---|---|---|
| Predictor | Coefficient (b) | SE | CI | *p*-value |
| intercept | 26.59 | 15.16 | [2.88, 62.29] | **0.020** |
| Age | -0.47 | 0.19 | [-0.92, -0.16] | **<0.001** |
| rMEQ | -0.09 | 0.63 | [-1.40, 1.04] | 0.851 |
| **Social Sleep Restriction** (Pandemic-2 - Pandemic-1) | | | | |
| Predictor | Coefficient (b) | SE | CI | *p*-value |
| intercept | 51.17 | 17.07 | [13.49, 85.57] | **0.010** |
| Age | -0.59 | 0.21 | [-.98, -0.14] | **<0.001** |
| rMEQ | -1.11 | 0.90 | [-2.92, 0.55] | 0.134 |
